# Supplementary figures and images for: Comprehensive Analysis of Human Subtelomeres by Whole Genome Mapping
Source: PLoS Genet. 2020 Jan 27;16(1):e1008347. doi: 10.1371/journal.pgen.1008347 (PMC7004388; doi:10.1371/journal.pgen.1008347)

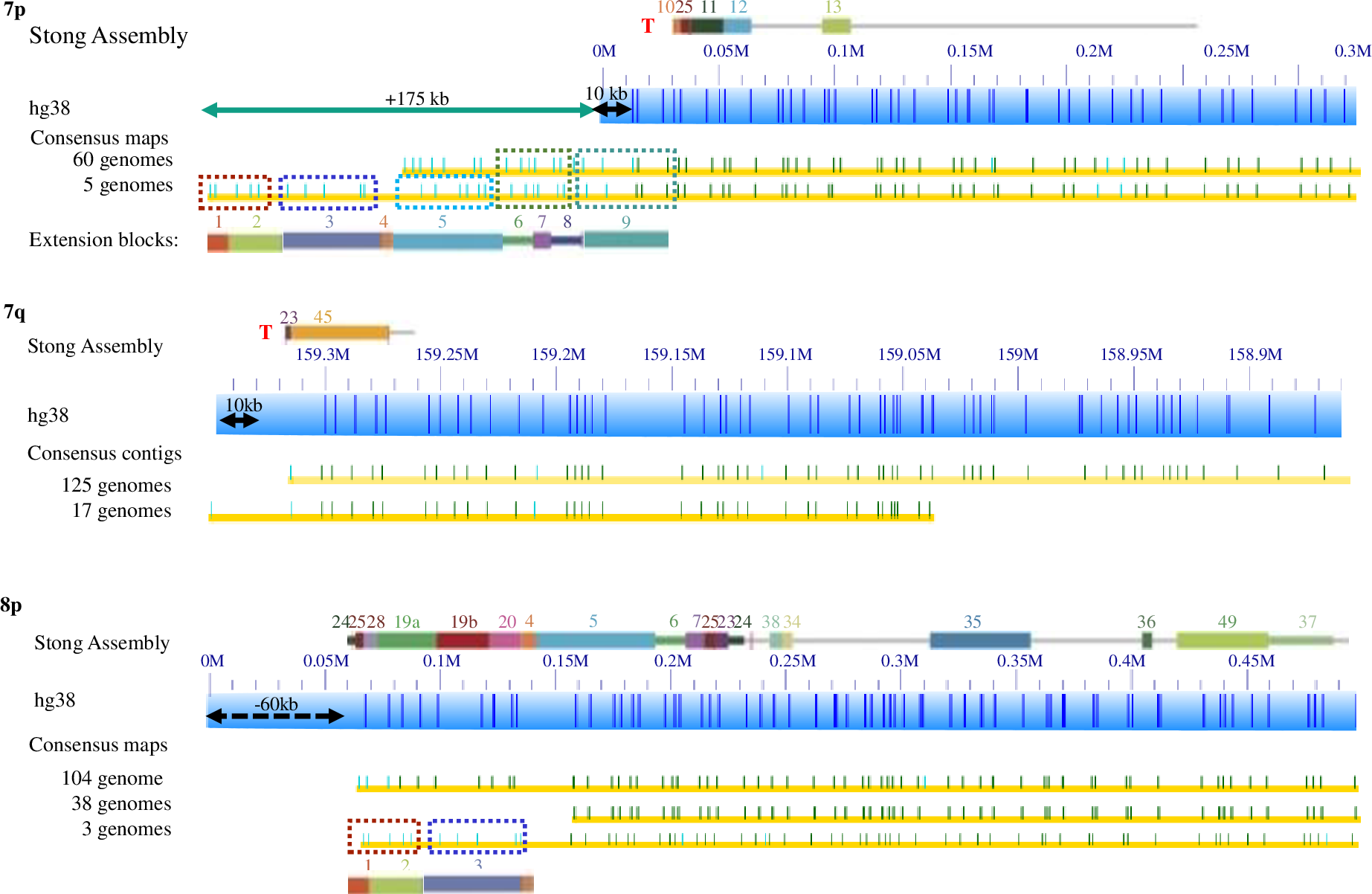

Supplement: S1 Fig — shows the major haplotypes for additional chromosome arms 7p, 7q and 8p in the highly variable set of subtelomeres.). The Stong Assembly paralogy blocks are shown as colored rectangles above blue Bionano optical mapping bars. Yellow rows with green ticks show haplotypes below these. A teal arrow indicates the size of additional extended regions not covered by the reference. A black arrow indicates the region indicated as a telomere-adjacent gap in the HG38 reference sequence. If the black arrow is dashed it signifies a region that should be deleted. (TIF) [file pgen.1008347.s001.tif]

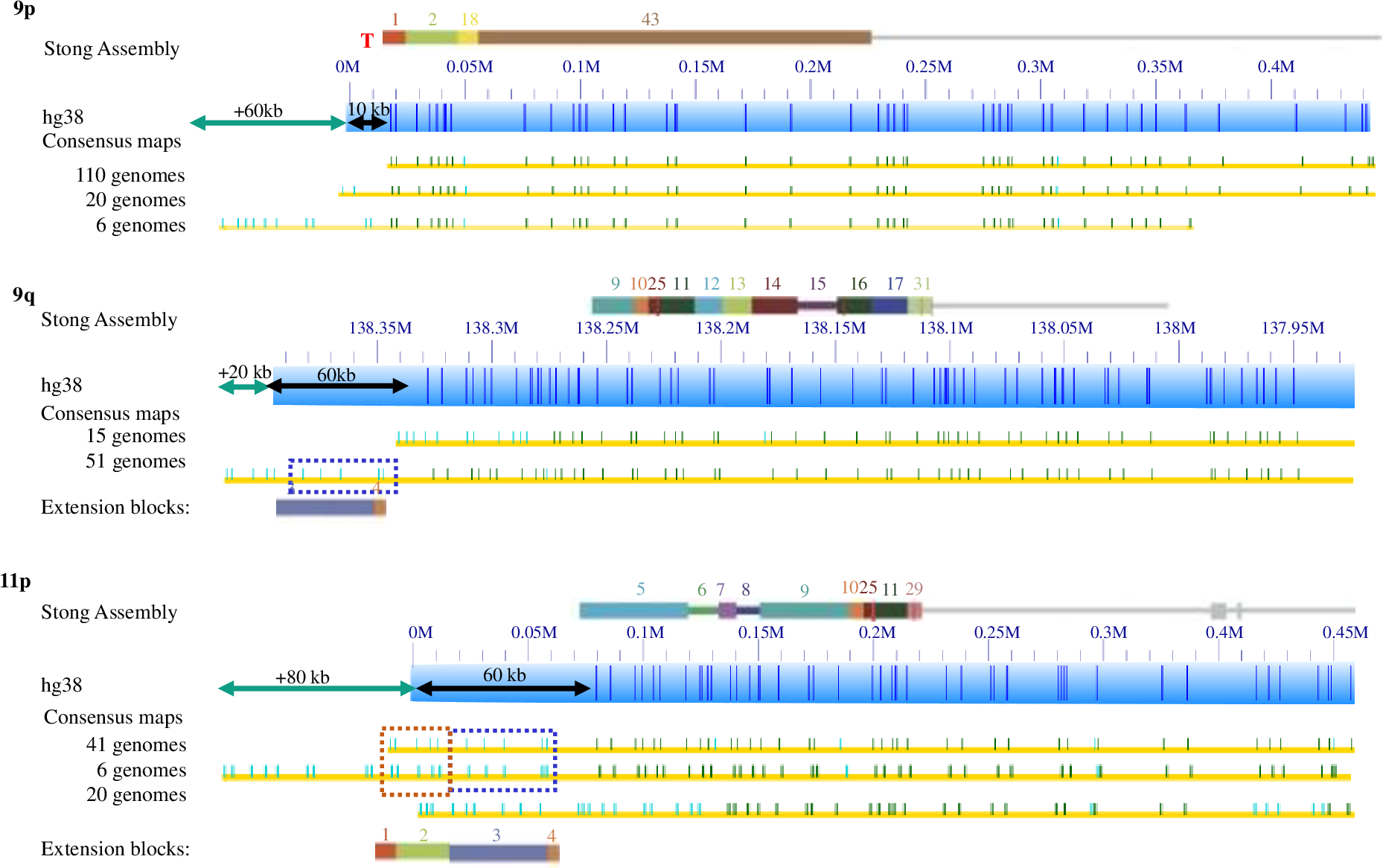

Supplement: S2 Fig — Chromosome arms 9p, 9q and 11p are shown. The Stong Assembly paralogy blocks are shown as colored rectangles above blue Bionano optical mapping bars. Yellow rows with green ticks show haplotypes below these. A teal arrow indicates the size of additional extended regions not covered by the reference. A black arrow indicates the region indicated as a telomere-adjacent gap in the HG38 reference sequence. If the black arrow is dashed it signifies a region that should be deleted. (TIF) [file pgen.1008347.s002.tif]

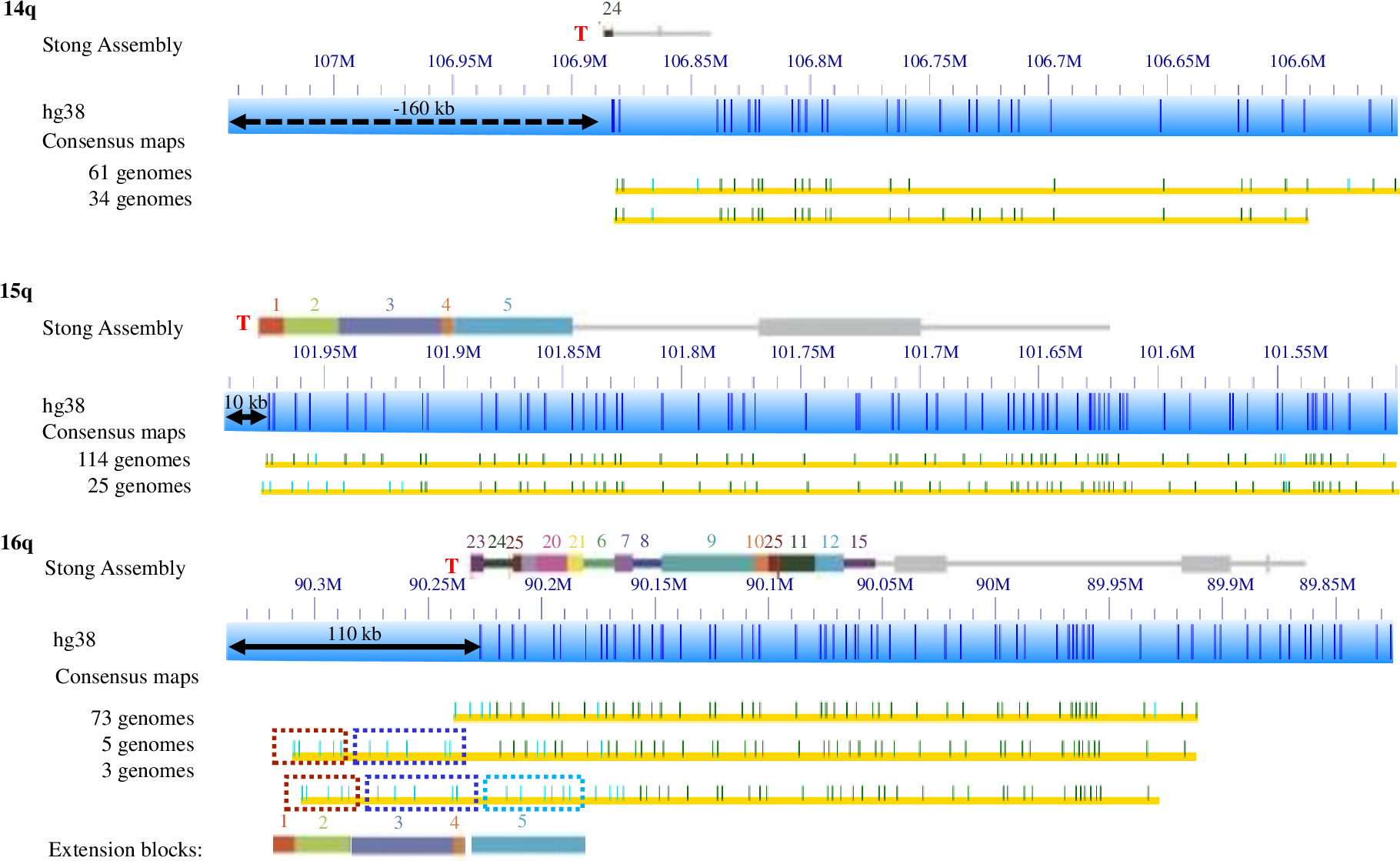

Supplement: S3 Fig — Chromosome arms 14q, 15q and 16q are shown. The Stong Assembly paralogy blocks are shown as colored rectangles above blue Bionano optical mapping bars. Yellow rows with green ticks show haplotypes below these. A teal arrow indicates the size of additional extended regions not covered by the reference. A black arrow indicates the region indicated as a telomere-adjacent gap in the HG38 reference sequence. If the black arrow is dashed it signifies a region that should be deleted. (TIF) [file pgen.1008347.s003.tif]

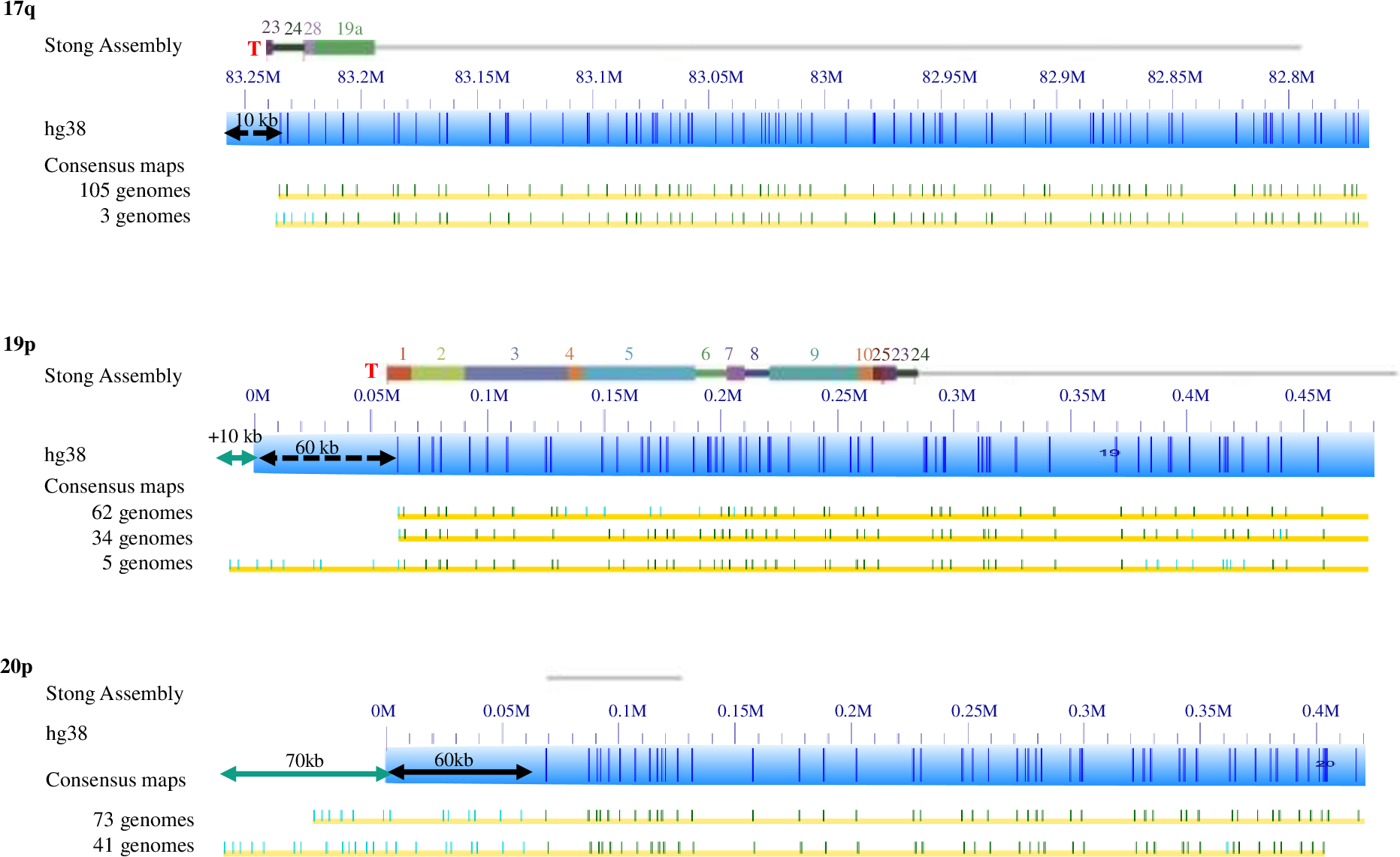

Supplement: S4 Fig — Chromosome arms 17q, 19p and 20p are shown. The Stong Assembly paralogy blocks are shown as colored rectangles above blue Bionano optical mapping bars. Yellow rows with green ticks show haplotypes below these. A teal arrow indicates the size of additional extended regions not covered by the reference. A black arrow indicates the region indicated as a telomere-adjacent gap in the HG38 reference sequence. If the black arrow is dashed it signifies a region that should be deleted. (TIF) [file pgen.1008347.s004.tif]

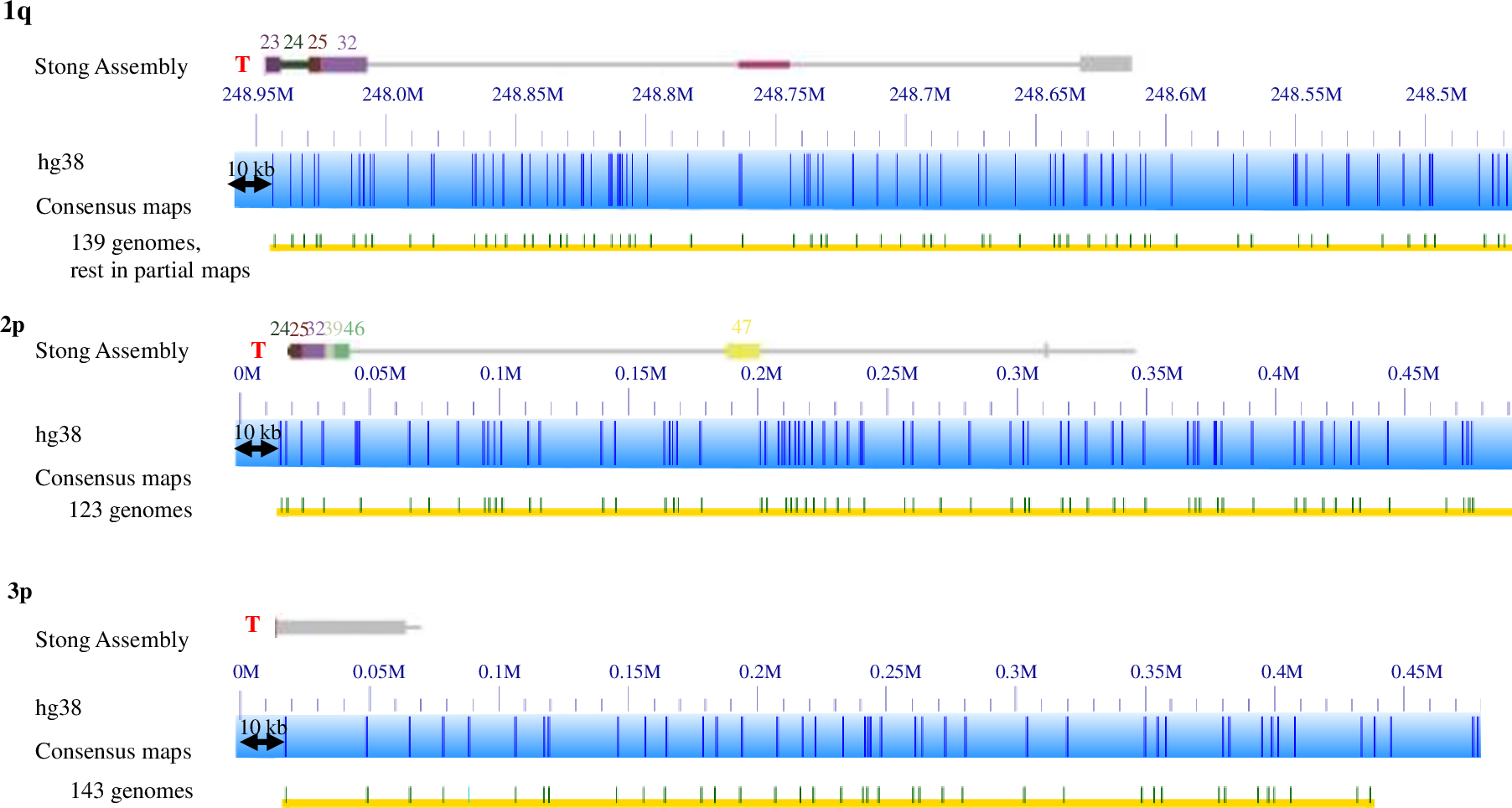

Supplement: S5 Fig — shows the major haplotypes for chromosome arms 1q, 2p and 3p in the less variable set of subtelomeres. The Stong Assembly paralogy blocks are shown as colored rectangles above blue Bionano optical mapping bars. Yellow rows with green ticks show haplotypes below these. A teal arrow indicates the size of additional extended regions not covered by the reference. A black arrow indicates the region indicated as a telomere-adjacent gap in the HG38 reference sequence. If the black arrow is dashed it signifies a region that should be deleted. Each low-variability arm (S5-S10) is briefly described in S1 Text. (TIF) [file pgen.1008347.s005.tif]

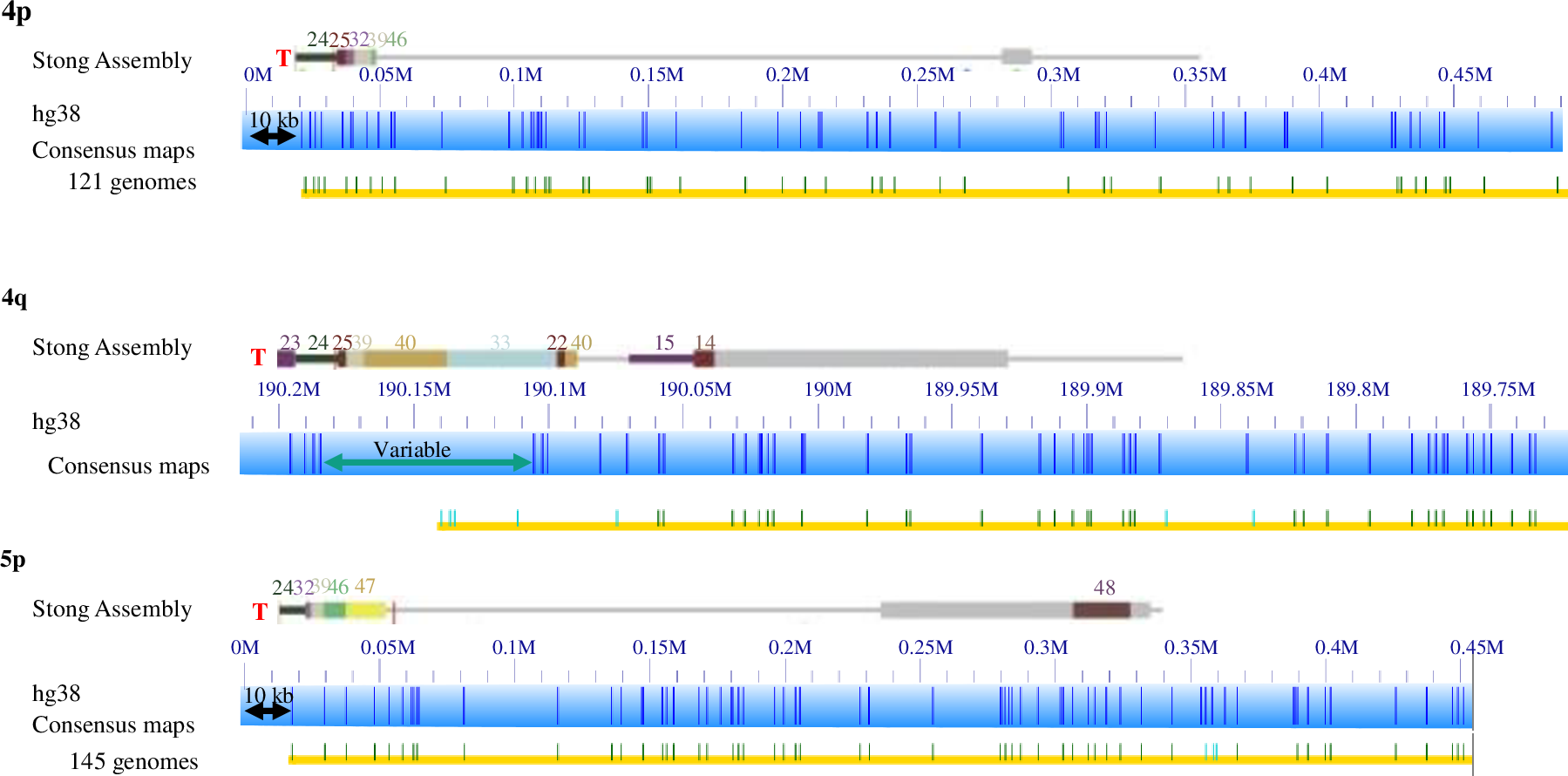

Supplement: S6 Fig — shows the major haplotypes for chromosome arms 4p, 4q and 5p in the less variable set of subtelomeres. The Stong Assembly paralogy blocks are shown as colored rectangles above blue Bionano optical mapping bars. Yellow rows with green ticks show haplotypes below these. A teal arrow indicates the size of additional extended regions not covered by the reference. A black arrow indicates the region indicated as a telomere-adjacent gap in the HG38 reference sequence. If the black arrow is dashed it signifies a region that should be deleted. (TIF) [file pgen.1008347.s006.tif]

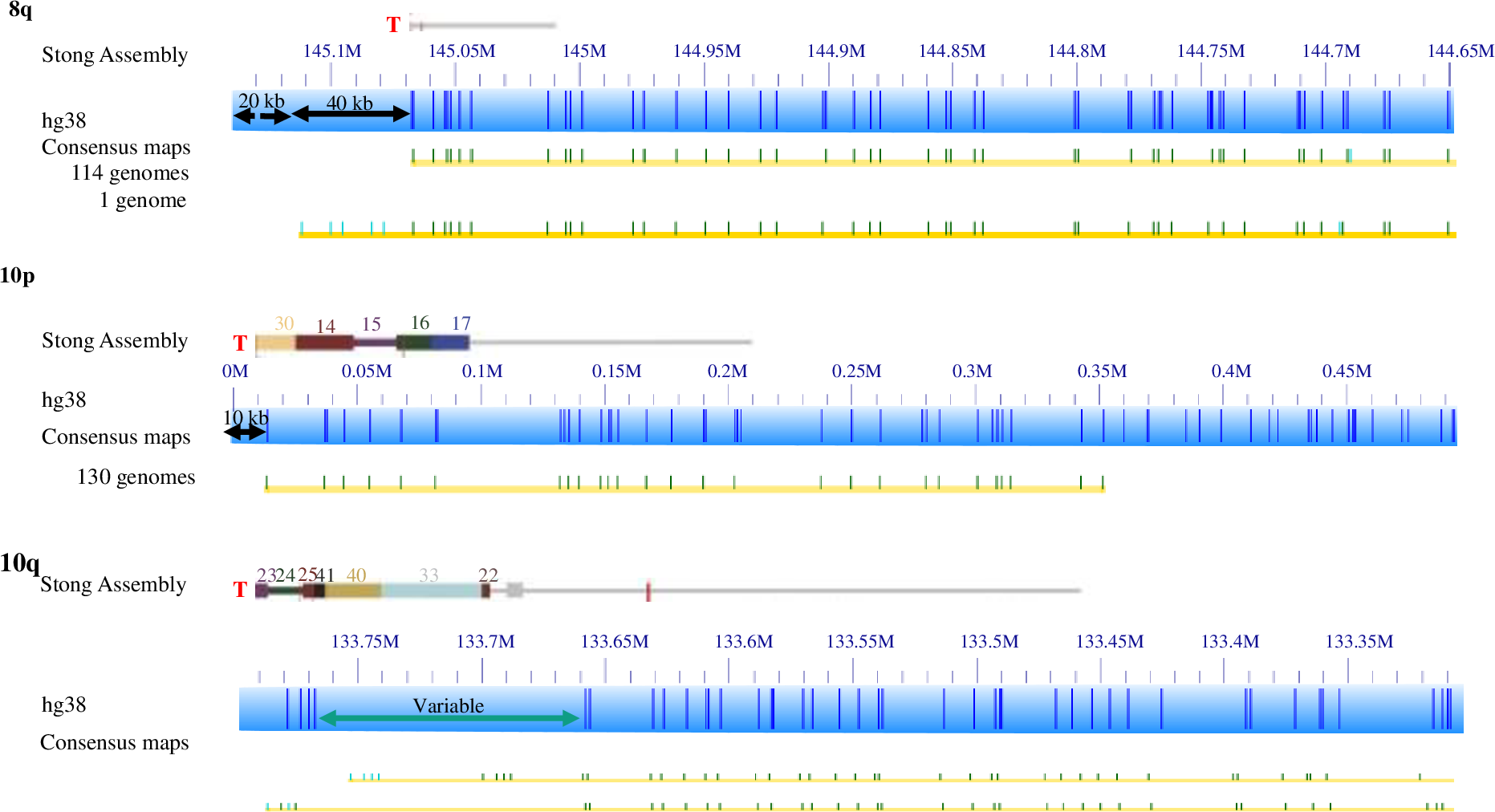

Supplement: S7 Fig — shows the major haplotypes for chromosome arms 8q, 10p and 10q in the less variable set of subtelomeres. The Stong Assembly paralogy blocks are shown as colored rectangles above blue Bionano optical mapping bars. Yellow rows with green ticks show haplotypes below these. A teal arrow indicates the size of additional extended regions not covered by the reference. A black arrow indicates the region indicated as a telomere-adjacent gap in the HG38 reference sequence. If the black arrow is dashed it signifies a region that should be deleted. (TIF) [file pgen.1008347.s007.tif]

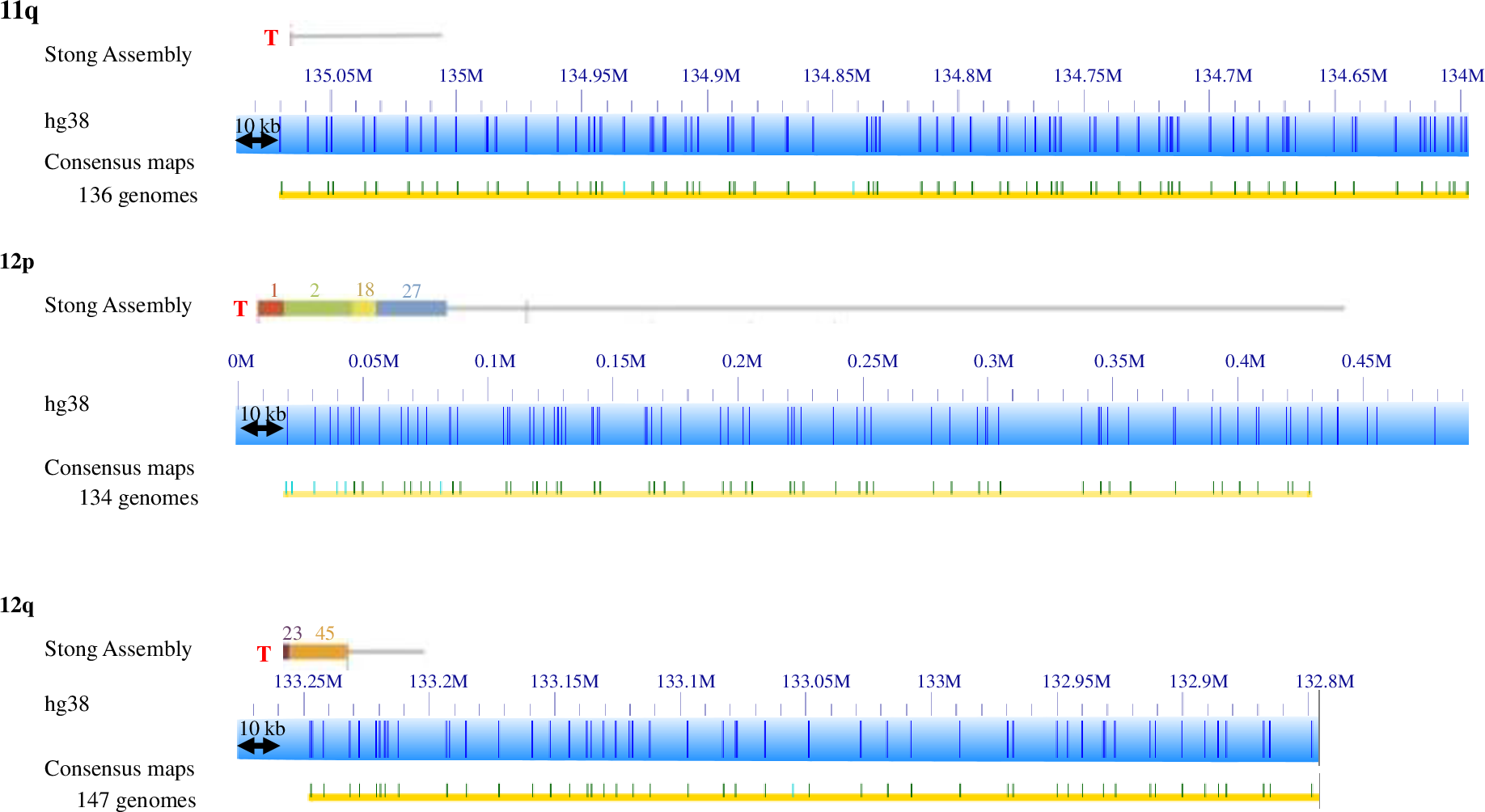

Supplement: S8 Fig — shows the major haplotypes for chromosome arms 11q, 12p, and 12q in the less variable set of subtelomeres. The Stong Assembly paralogy blocks are shown as colored rectangles above blue Bionano optical mapping bars. Yellow rows with green ticks show haplotypes below these. A teal arrow indicates the size of additional extended regions not covered by the reference. A black arrow indicates the region indicated as a telomere-adjacent gap in the HG38 reference sequence. If the black arrow is dashed it signifies a region that should be deleted. (TIF) [file pgen.1008347.s008.tif]

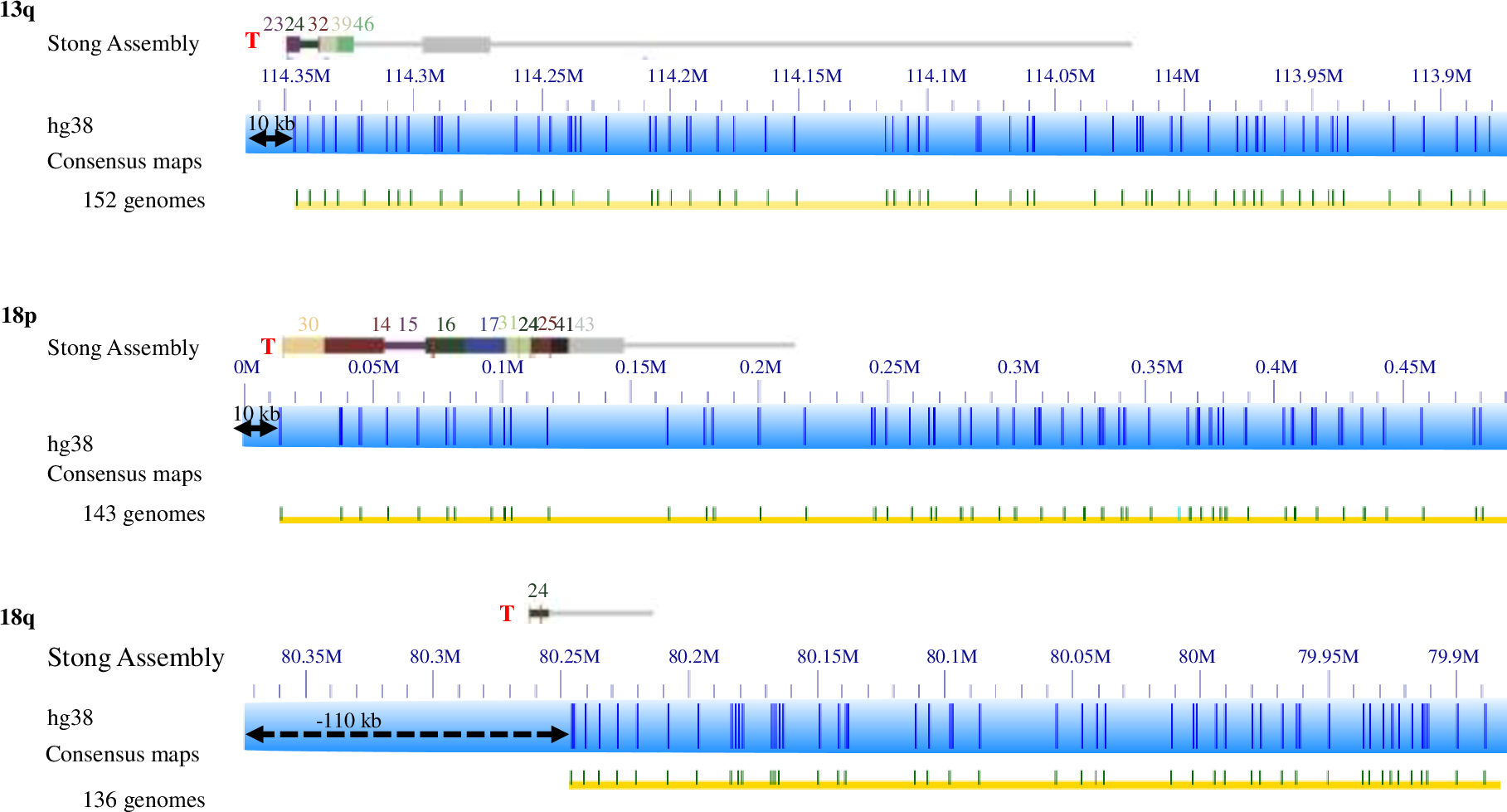

Supplement: S9 Fig — shows the major haplotypes for chromosome arms 13q, 18p, and 18q in the less variable set of subtelomeres. The Stong Assembly paralogy blocks are shown as colored rectangles above blue Bionano optical mapping bars. Yellow rows with green ticks show haplotypes below these. A teal arrow indicates the size of additional extended regions not covered by the reference. A black arrow indicates the region indicated as a telomere-adjacent gap in the HG38 reference sequence. If the black arrow is dashed it signifies a region that should be deleted. (TIF) [file pgen.1008347.s009.tif]

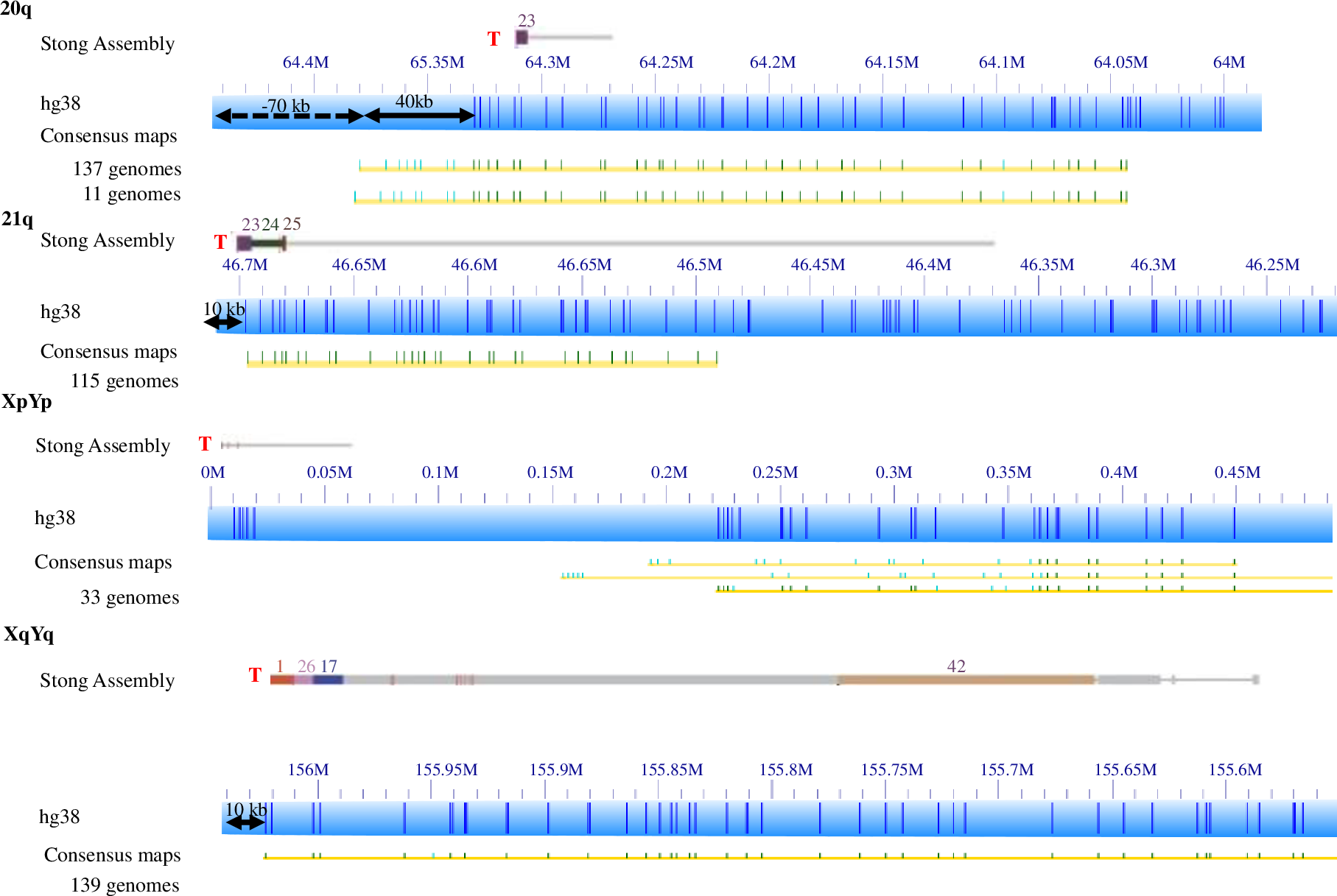

Supplement: S10 Fig — shows the major haplotypes for chromosome arms 20q, 21q, XpYp and XqYq in the less variable set of subtelomeres. The Stong Assembly paralogy blocks are shown as colored rectangles above blue Bionano optical mapping bars. Yellow rows with green ticks show haplotypes below these. A teal arrow indicates the size of additional extended regions not covered by the reference. A black arrow indicates the region indicated as a telomere-adjacent gap in the HG38 reference sequence. If the black arrow is dashed it signifies a region that should be deleted. (TIF) [file pgen.1008347.s010.tif]

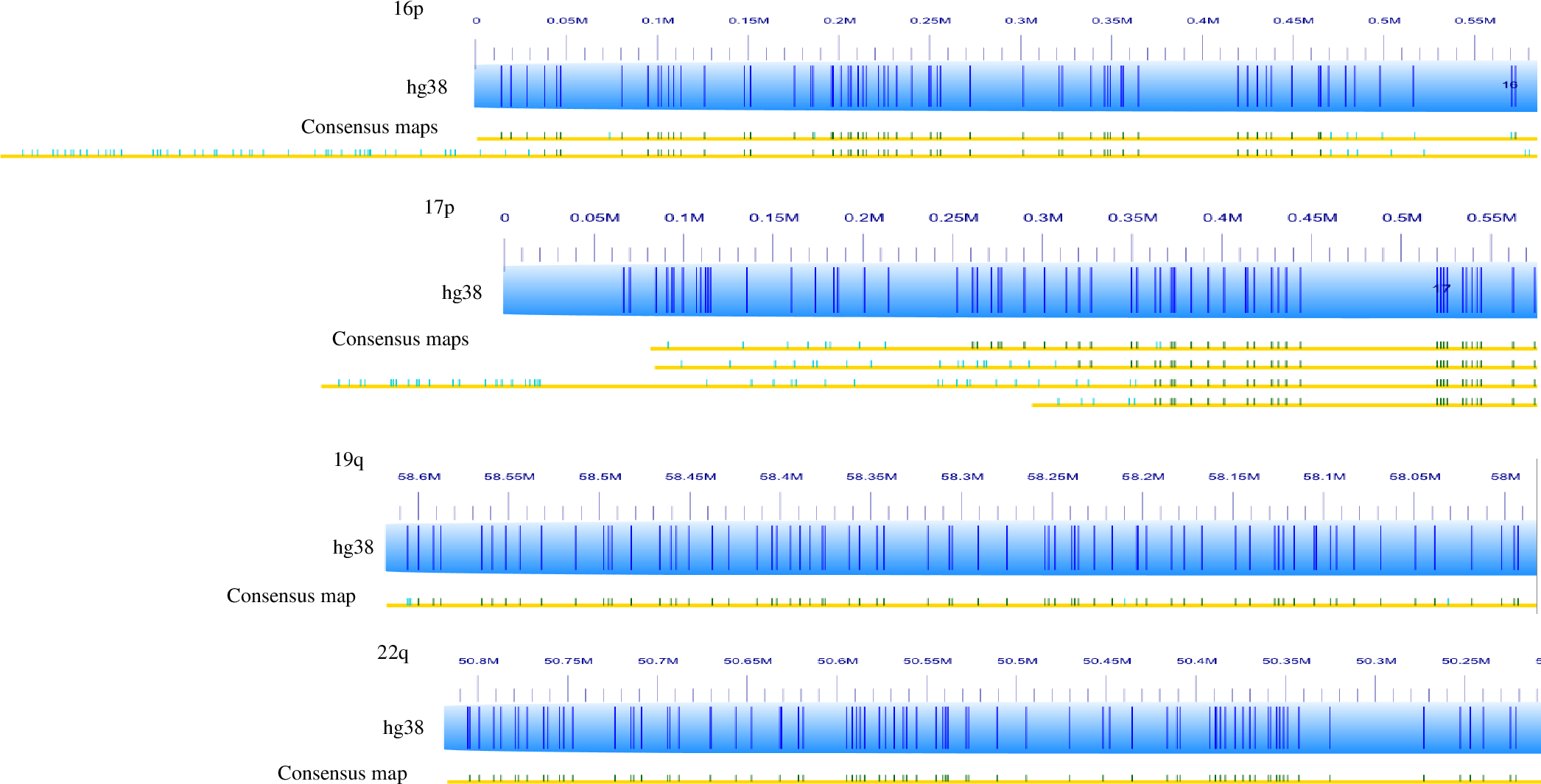

Supplement: S11 Fig — Reference and consensus maps for arms 16p, 17p, 19q, and 22q are shown. Blue bars with dark blue lines indicate the location of labels in HG38. Yellow rows with green ticks show haplotypes below these for each arm. (TIF) [file pgen.1008347.s011.tif]
