# Supplementary material for: Comprehensive Analysis of Human Subtelomeres by Whole Genome Mapping
Source: PLoS Genet. 2020 Jan 27;16(1):e1008347. doi: 10.1371/journal.pgen.1008347 (PMC7004388; doi:10.1371/journal.pgen.1008347)
Supplement: S1 Text — (DOCX) [file pgen.1008347.s013.docx]

S1 Text: Descriptions of haplotypes in low variability arms

**1q:** 139 genomes have the same haplotype, matching well to the existing hg38 reference. 5 genomes start 248.93Mb and DNA from the remainder (10 genomes) failed to assemble a contig at this telomere.

**2p**: 123 genomes have exactly the existing hg38 reference. 5 are the same but have an additional nick site 0.02Mb past these 123. 2 have a different pattern but covering the same length at the majority, and this unknown pattern doesn’t match any additional blocks. DNA from 24 genomes failed to assemble a contig at this telomere.

**3p**: Arm 3p is almost identical for all genomes, 143 have contigs matching the reference. The Stong assembly shows no paralogy blocks for this arm. DNA from 11 genomes failed to assemble a contig at this telomere.

**4p**: 121 genomes match each other and the HG38 reference. 3 genomes contain a single additional nick site adjacent to the final one. DNA from the remaining 30 genomes failed to assemble a contig at this telomere.

**4q**: The 4q arm contains a wide range of haplotypes that unlike most arms do not show differences in nicking patterns. Instead, the variation here is in the length of the region starting at 190.06Mb, and ending anywhere from 190.15Mb to 190.36Mb. This is due to a known variation in the number of copies of the D4Z4 repeat (31, 32).

**5p**: 145 genomes have a haplotype matching well to the HG38 reference. 7 have a 5kb narrower spacing of the last few nick sites. DNA from 2 genomes failed to assemble a contig at this telomere.

**8q:** 114 genomes have an 8q haplotype matching the reference accurately. 1 sample has an additional 0.04Mb. DNA from 36 genomes failed to assemble a contig at this telomere.

**10p**: 130 of genomes match the HG38 reference precisely. DNA from 24 genomes failed to assemble a contig at this telomere.

**10q**: The 10q arm, like 4q, has an interesting situation where the nick site patterns show consistency with the reference but a variation in length between the distal-most and the more centromeric sets of nick sites. This is due to the D4Z4 repeat (3.3kb) located starting after 133.66Mb and continuing until close to the end of the arm. The number of copies of the repeat varies, with healthy individuals having more than 11 copies (31). This causes the length of the arm to vary, our data set shows the last nick site of 10q ranging from 133.70Mb to 133.85Mb.

**11q:** 137 genomes agree with the HG38 reference for 11q. DNA from 17 genomes failed to assemble a contig at this telomere.

**12p**: 134 genomes match with the HG38 reference. 11 genomes fall into 1 of four haplotypes which match the length of the majority haplotype but have some minor differences from 0.02Mb to 0.04Mb. DNA from 9 genomes failed to assemble a contig at this telomere.

**12q**: 147 arms have a haplotype matching the current reference. DNA from 7 genomes failed to assemble a contig at this telomere.

**13q**: 152 of genomes have sequences matching HG38. DNA from 2 genomes failed to assemble a contig at this telomere.

**18p**: 143 of the genomes match the HG38 reference precisely. 1 contains a 0.26Mb extension. DNA from 7 genomes failed to assemble a contig at this telomere.

**18q**: 136 of the mapped genomes contigs end at 80.25Mb and match well to the reference. 14 are missing the distal portion compared to the majority haplotype and start at 80.15Mb. HG38 show a large telomere-adjacent gap, which is incorrect according to our data, indicated by a dashed black line. DNA from 4 genomes failed to assemble a contig at this telomere.

**20q**: Arm 20q extends into the telomere adjacent gap Ns for almost all of its haplotypes. 137 of genomes have a pattern with a 0.05Mb extension. 11 of genomes have one of three variations on this haplotype’s pattern. DNA from 6 genomes failed to assemble a contig at this telomere.

**21q**: 115 genomes match the HG38 reference, starting at 46.5Mb and ending at 46.7Mb. DNA from 39 genomes failed to assemble a contig at this telomere.

**XpYp**: Only a few genomes (33) had subtelomeric contigs for this region. They have similar patterns but end at different lengths, none close to the telomere.

**XqYq**: 139 genomes matched well to the reference for this arm. DNA from 15 genomes failed to assemble a contig near this telomere.

**16p, 17p, 19q and 22q**: These arms contain an inverted nick-pair (INP) site for the nicking enzyme Nt.BspQ1 in the subtelomeric region that precluded their initial analysis. An alternative labeling method confirmed interference by this INP site, and suggested that 19q and 22q have very low variation while 16p and 17p may have high levels of structural variation. (See main text).

No mapped assemblies for the acrocentric short-arm subtelomeres (22p, 21p, 13p, 14p, 15p) are available since this sequence is missing from the HG38 reference.
